# A Large Extragnathic Keratocystic Odontogenic Tumour

**DOI:** 10.1155/2015/723010

**Published:** 2015-12-06

**Authors:** Soumya Makarla, Radhika M. Bavle, Sudhakara Muniswamappa, Srinath Narasimhamurthy

**Affiliations:** ^1^Department of Oral Pathology, Krishnadevaraya College of Dental Sciences, Bangalore 562157, India; ^2^Department of Oral Surgery, Krishnadevaraya College of Dental Sciences, Bangalore 562157, India

## Abstract

Odontogenic keratocysts (OKCs) are developmental cysts which occur typically in the jawbones. They present more commonly in the posterior mandible of young adults than the maxilla. OKCs have been reclassified under odontogenic tumours in 2005 by the WHO and have since been termed as keratocystic odontogenic tumours (KCOTs). Here we report a case of a recurrent buccal lesion in a 62-year-old man which was provisionally diagnosed as a space infection (buccal abscess) but surprisingly turned out to be a soft tissue KCOT in an unusual location on histopathologic examination.

## 1. Introduction

Odontogenic keratocysts are developmental odontogenic cysts which were reclassified as tumours in 2005 by the WHO on account of their aggressive behavior and recurrence potential [1]. They are most commonly gnathic lesions, meaning that they arise within the jaws, that is, the mandible and maxilla. Occasionally they have been found to arise in a peripheral location especially in the gingiva which has earned them the term “peripheral KCOTs” [1].

The histogenesis of intraosseous KCOT can be attributed to the sources of odontogenic epithelium, that is, dental lamina and its remnants and the extensions of basal cells from the overlying epithelium [2]. The involvement of the Hedgehog signaling pathway due to PTCH-1 inactivation has been suggested and accepted as the underlying cause in the pathogenesis of this cystic tumour [2].

Cysts and tumours which arise from the epithelium and its remnants associated with tooth development are termed as “odontogenic.” These lesions typically occur centrally within the bone. However, occasional reports exist concerning those odontogenic lesions having occurred in the soft tissues overlying the alveolar bone. These have been aptly termed “peripheral” or “extragnathic” [3].

Rare cases of parakeratinized cysts in the facial soft tissues, skin, muscles of mastication, and also temporomandibular joint, which histopathologically resemble intraosseous KCOT, have been reported. Owing to the rarity of these loci, variants of KCOT can be described and further discussed as intraosseous, solid-cystic, and peripheral types [2].

## 2. Case Report

A 62-year-old male reported to our hospital with an asymptomatic swelling in the right lower half of the face with reduced mouth opening since two weeks. History revealed that this was the 3rd time that such a swelling had appeared in the buccal region over a span of 8 years. The first episode 5 years before involved a previous history of pus discharge with a diagnosis of buccal space infection treated by incision and drainage. The buccal swelling had regressed on its own upon a second occurrence 2 years back. The patient has been a known diabetic since a year and on regular medication. A detailed medical examination was performed in which the patient was screened for anomalies of the skin, eyes, skeletal system, endocrine system, nervous system, and any other developmental disorders, ruling out any syndromic association.

**Figure 1:**
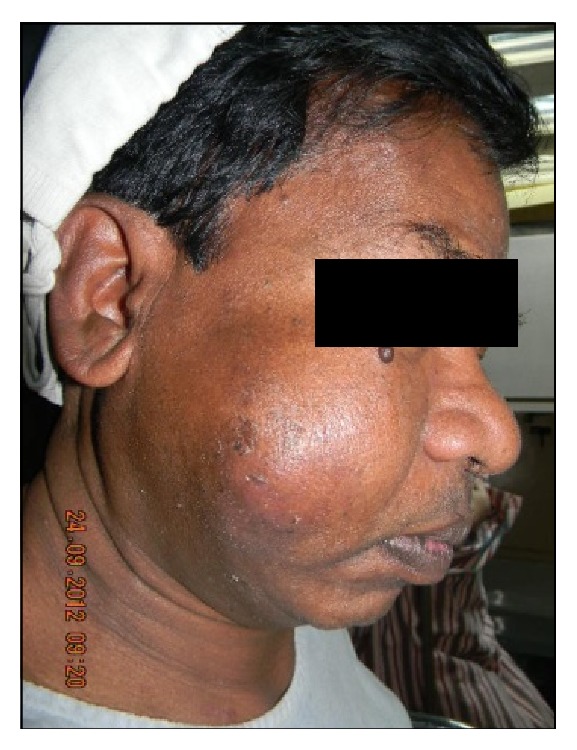
Clinical image showing right side of the face with a solitary mass extending from posterior ala region to preauricular area. Superiorly, it extends till infraorbital margin to angle of the mouth inferiorly.

On examination, gross asymmetry on the right side of the face was evident. A large ovoid submucosal swelling (6 × 6 cm in size) was observed extraorally, extending superoinferiorly from the level of the right lateral canthus of the eye to 1 cm below the corner of the mouth and anteroposteriorly from the corner of the mouth to the mandibular ramus (Figure 1). The borders of the lesion were ill-defined on palpation. The consistency of the swelling varied from being soft to firm. Overlying skin was normal. There was no history of any pain, fever associated with the swelling. Submandibular and cervical lymphadenopathy was absent. Paresthesia or palsy of the right side of the face or swelling of the right parotid gland was not noted.

Intraorally, the ovoid swelling presented as a slight elevation of the buccal mucosa without any obliteration of the buccal vestibule. It extended from 1 cm from the commissure of lip to 1 cm short of the pterygomandibular raphae. The buccal mucous membrane appeared stretched and glistening without any signs of inflammation. Mouth opening was restricted due to the size of the lesion.

**Figure 2:**
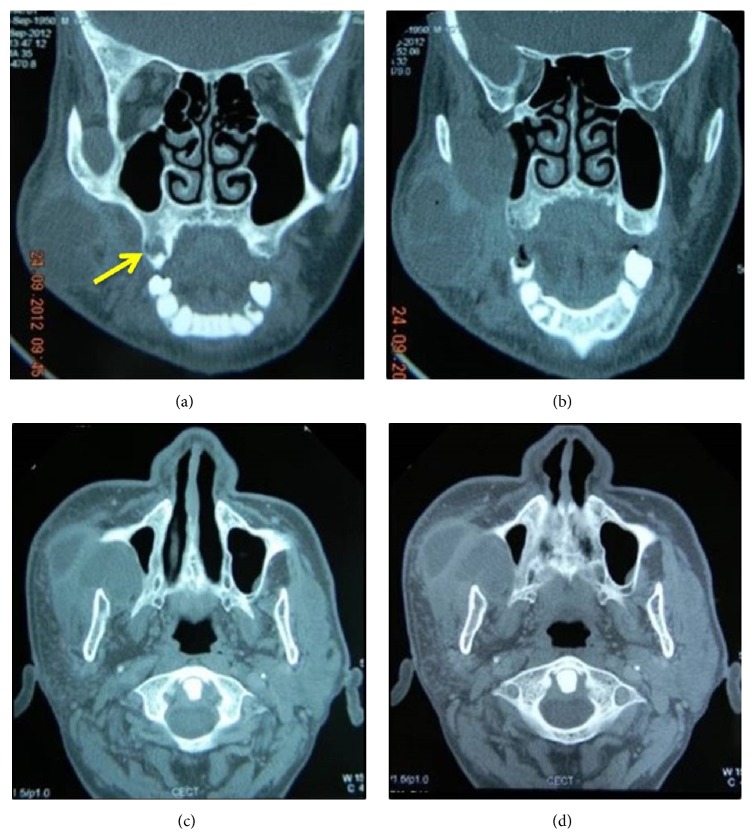
((a) and (b)) Posteroanterior view; ((c) and (d)) transverse CT at C 6 level: CT scan showing a well-defined loculated soft tissue mass in the buccal space. The lesion shows multilocularity with gel or fluidic content. The lesion is seen compressing the maxillary bone in the area of antral wall laterally.

Computed tomography revealed a well-defined lesional mass with cystic areas involving the buccal space. Intracystic loculation was visible with a pus-like dense cystic content. Erosion of the posterior buccal cortex was evident with no perforation of the maxillary bone. The maxillary antral wall was compressed (Figures 2(a)–2(d)).

**Figure 3:**
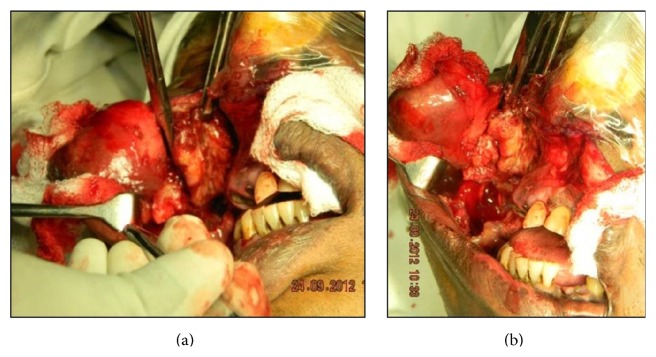
((a) and (b)) Surgical image showing a capsulated enmasse of lesion which was easily dissected out, devoid of any muscle or bone involvement.

A clinical diagnosis of a nonspecific chronic abscess with spread of infection was made and the lesion was treated under GA through an intraoral approach. An incision was made on the buccal mucosa and the lesion was excised in toto along with a safe margin of a fibrous tissue cuff. The lesion shelled out easily without any involvement of the buccinator muscle nor of any bony component. On surgical exposure, the lesional mass was well circumscribed with a fibrous outer covering. The lesion looked lobulated as the mass had herniated into the pterygomaxillary space (Figures 3(a) and 3(b)).

The excised lesion resembling a leathery pouch was present completely within the soft tissue in the buccal space. It was not attached to the buccinator muscle nor was it attached to any bony component of the maxilla or related to the parotid gland or duct.

**Figure 4:**
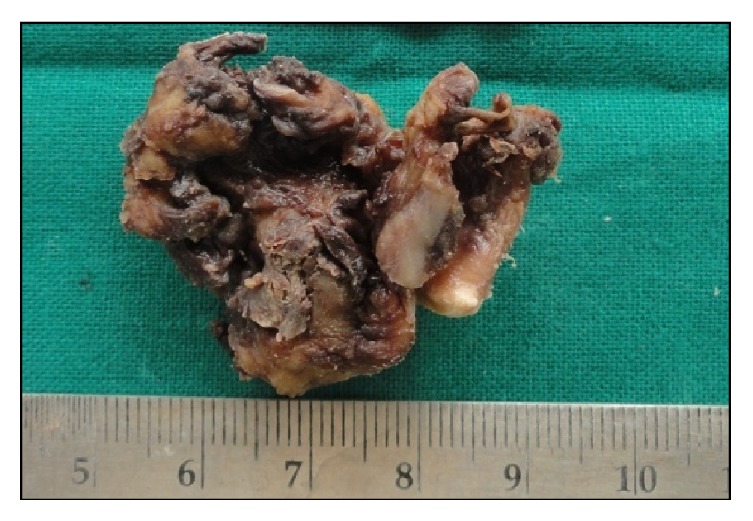
Gross image of a 4.5 × 4.8 cm lobulated soft tissue mass.

The specimen obtained was 4.8 × 4.5 cm in size, creamish brown in colour with a firm consistency with an irregular surface. Multiple samples were routinely processed and stained with hematoxylin and eosin (Figure 4).

A differential of a large dermoid or epidermoid cyst, a parasitic cyst, and a cyst of inflammatory origin was considered.

**Figure 5:**
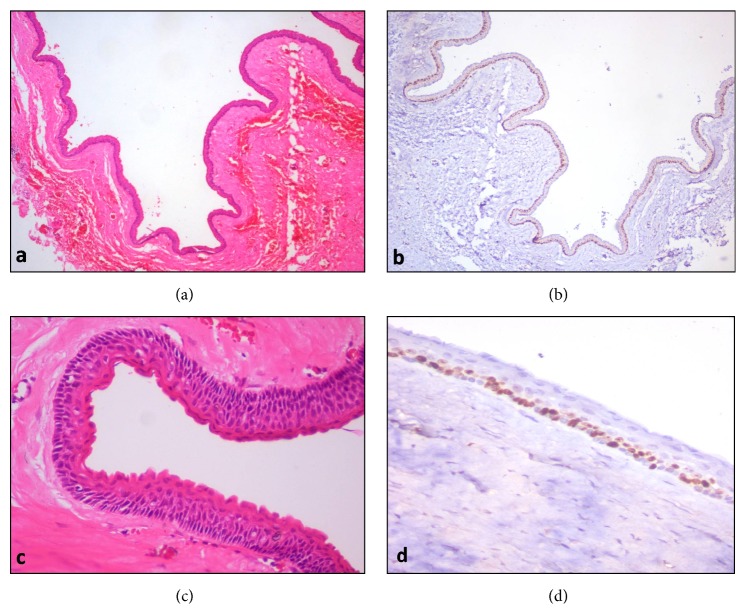
(a) Hematoxylin and eosin stained histopathological section of the lesion showing cystic epithelium of odontogenic keratocyst with numerous infoldings (×40). (b) Corresponding IHC image showing nuclear expression of Ki-67 seen continuously in the basal layer (×40). (c) High power view showing 5–7 cell layer thick, corrugated parakeratinized cystic epithelium with columnar basal cells exhibiting palisading nuclear polarity (H&E stain, ×200). (d) Corresponding IHC image showing nuclear expression of Ki-67 continuously in the basal layer and sparsely in the parabasal layer. Superficial layers are completely free of Ki-67 expression (×200).

Microscopically, the tissue sections showed a cystic lumen lined by parakeratinized epithelium of uniform thickness. The basal cells were cuboidal to columnar in shape exhibiting nuclear palisading. The spinous layer was thin and the parakeratin layer showed surface corrugations. Some areas were lined by flattened, stretched epithelium whereas other areas exhibited numerous infoldings of the epithelium into the connective tissue wall. The epithelium connective tissue interface was smooth and flat with no rete ridges. Underlying fibrous connective tissue was thin and mature with fibrocytes and fibroblasts with few odontogenic rests. Areas of haemorrhage and blood vessels were evident in the stroma. No daughter cysts, epithelial islands, skeletal muscle tissue, or any appendages were present in the connective tissue wall (Figures 5(a) and 5(c)).

A diagnosis of an* extragnathic KCOT of the buccal region* was made taking into account mainly the histologic features supported by the clinical and CT (radiologic features). Regular patient follow-up is carried out every 6 months with no evidence of recurrence in 24 months.

The lesional tissue was immunostained with Ki-67 (Biogenex) to assess the mitotic activity of the cystic lining to reveal the proliferative potential of the lesion as compared to conventional intraosseous KCOT. An interesting finding was that almost all the basal cells and only few suprabasal cell nuclei stained positive for Ki-67. A converse of this finding is generally observed in most conventional intraosseous KCOTs wherein a larger number of suprabasal cell nuclei stain positive for Ki-67 as opposed to the nuclei of the palisading basal cells (Figures 5(b) and 5(d)).

## 3. Review of Literature

A PubMed search of the English literature of all the cases of KCOT occurring in the buccal region has been compiled in a chronological order in Table 1.

## 4. Discussion

KCOTs are cystic odontogenic lesions arising in the gnathic skeleton. KCOT has been defined by the latest WHO classification of tumours of head and neck as “a benign uni- or multicystic, intraosseous tumour of odontogenic origin, with characteristic lining of parakeratinized stratified squamous epithelium and potential aggressive, infiltrative behaviour” [6]. KCOTs have a distinct pattern of growth, that is, by traversing through the marrow spaces of the jaws, and also have a high recurrence rate of 23.15% [7].

Though KCOTs conventionally occur in an intraosseous location; reports of odontogenic tumours like ameloblastoma, CEOT, and AOT including KCOT arising in a peripheral location exist in literature [8]. The location of these peripheral counterparts has most commonly been in the gingiva. Therefore the term “peripheral” has come to depict a primarily intraosseous lesion occurring in the gingival soft tissues.

However, KCOTs of the soft tissue have been reported in other anatomic sites like the buccal mucosa and masticatory muscles apart from the gingiva. A review of the English literature revealed 25 cases of peripheral (soft tissue) KCOTs. Of the 25, 17 of them were found in the gingiva, 6 in the buccal mucosa, and 2 cases intramuscularly [1], the details of which are listed in Table 1. Therefore the terms “extraosseous” or “extragnathic” would be more apt in referring to those lesions occurring in the soft tissues other than gingiva. Ours will hence be the 7th reported case of a soft tissue KCOT occurring in the buccal mucosa and the 26th case of a peripheral KCOT [2, 9].

Generally KCOTs of the jawbone do not produce a large expansion of the cortex as the cyst proliferates within the marrow spaces, thus growing in a longitudinal manner. But when it occurs in the buccal soft tissues, it tends to grow in a centrifugal direction, therefore causing the cyst to expand uniformly on all sides, resulting in a large buccal swelling as the main clinical presentation. The cyst also simulated spread of infection as it had extended into the pterygomandibular space and infratemporal space but could be easily dissected during surgery.

Plain radiographs play a very limited role in the detection of a soft tissue lesion. However, studies prove that the positive rate of computed tomography (CT) in the diagnosis of PKCOT (peripheral KCOTs) is 100%. CT can reveal the relationship between the lesion and the adjacent hard or soft tissues. By measuring the CT value, the lesion can be defined as cystic [10]. These features concur with the CT findings in our case of a well-defined cystic mass involving the buccal space. Intracystic loculation could also be appreciated with cystic content. The bony findings of erosion of the posterior buccal cortex with no perforation of the maxillary bone also provided more clarity in diagnosing the lesion being situated in the buccal space.

Though an organising abscess with buccal space infection can give a similar picture, absence of signs of inflammation ruled out this possibility.

KCOT is a distinct lesion, owing to its unique histopathology. The cystic lining is characterized by a parakeratinized stratified squamous epithelium of uniform thickness, with a corrugated surface and a palisaded and polarized basal layer with “picket fence” or “tombstone” appearance, devoid of rete ridges. Numerous infoldings of the epithelium are a common finding. The cystic lumen generally contains a straw colored fluid or a thick creamy material which may represent keratin [11].

Our case also exhibited a cystic epithelium comprising of numerous folds and a benign fibrous connective tissue wall. The lining was characterized by 5-6-cell layer uniformly thick epithelium with parakeratinization and surface corrugation, basal cell palisading and devoid of rete pegs which conforms to the typical descriptive diagnosis of an intraosseous KCOT.

The main histopathologic differentials for a soft tissue KCOT include epidermoid cyst, cutaneous keratocyst (CKC) ectopic occurrence of keratocyst of skin adnexa in buccal mucosa (hair follicles, sebaceous glands), trichilemmal cyst, and steatocystoma.

Epidermoid cysts are developmental pathologies occurring in the head and neck region almost always found in the regions of embryonic fusion with an intraoral incidence of less than 0.01% [12]. Histologically, epidermoid cysts reveal a cystic cavity filled with large amounts of keratin flakes lined by orthokeratinized stratified squamous epithelium with prominent stratum granulosum. Surface corrugations are seen in few areas which may resemble KCOT. KCOTs may be distinguished by the presence of parakeratinization and absence of a prominent stratum granulosum. Their basal cell layer shows prominent palisading nuclei which is lacking in an epidermoid cyst.

CKCs were first reported by Barr et al. as subcutaneous cysts in 1986 [13]. CKCs are highly correlated with nevoid basal cell carcinoma syndrome (NBCCS), 91.7% [13–16]. But there are reports of cases of CKCs having risen independent of the syndrome. They most commonly occur in the extremities and their cystic content resembles a brown grumous substance [17].

The lining epithelium comprises several layers of squamous epithelial cells without a granular cell layer and skin appendages. The cystic epithelium appears wavy or festooned with normal maturation [17]. Presence of mural daughter cysts is a common finding [9, 13]. Distinguishing these CKCs from KCOT is difficult as the corrugated anagen-like lining of these cysts resembles that of a KCOT. Therefore the possibility of a CKC arising ectopically in the buccal mucosa in such cases cannot be completely omitted [1].

Steatocystoma simplex is an uncommon benign cutaneous hamartomatous malformation arising from the pilosebaceous duct junction [17, 18]. It generally occurs on the trunk, axilla, scalp, and neck. Their cystic lumen consists of an oily sebaceous material [19]. Steatocystoma simplex is found as a solitary cystic nodule. The cyst exhibits folds of stratified squamous epithelium, basal layer palisading, and absence of granular layer with a characteristic dense eosinophilic hyaline layer/cuticle present on the epithelium. Lobules of sebaceous glands are evident in its connective tissue wall [20, 21]. It can be distinguished from KCOT by the presence of the flattened sebaceous structures.

Trichilemmal cysts mainly occur on the scalp and their cystic content comprises a cheesy, foul smelling thick material [17]. The cyst walls are composed of epithelial cells with no clearly distinguishable intercellular bridges. The peripheral cells show a distinct palisade arrangement. Epithelial cells close to the lumen are swollen and eosinophilic and a distinct granular layer is absent. The lumen is filled with eosinophilic homogenous material (trichilemmal keratinization) [22].

The mechanism of KCOT development in the soft tissues especially the buccal mucosa is yet to be established. It is common knowledge that the KCOT is considered to originate from the remnants of dental lamina. Therefore, to have occurred in the buccal soft tissues, these cells should have been displaced into the buccal mucosa and persisted during embryogenesis [23]. Recent reports may provide important clues in understanding the relationship between deciduous dentition and the oral vestibule during embryological development. According to them the primitive vestibular lamina involved in the formation of the future oral vestibule and the buccal mucosa reiteratively intermigrated with the dental epithelium around the maxillary molar areas during tooth formation [24, 25].

These findings suggest that the remnants of the dental lamina entrapped in the buccal tissues during embryogenesis may be triggered to form a keratocyst with the features of KCOT. Other odontogenic lesions such as ameloblastoma and odontoma developed in the buccal mucosa have also been reported in the English literature [26, 27].

It is also established that KCOTs may originate from the basal cells of the oral epithelium [4]. Therefore, the prospect of an extraosseous KCOT developing from the basal epithelial cell cannot be completely discarded. Surgical implantation of the cyst on account of the recurrent nature of the lesion should also be considered as a probable reason for occurrence.

In the statistics of soft tissue KCOT given by Abé et al., the recurrence rate of 21 cases was 13.6%, which is in close proximity to the recurrence rate of jawbone KCOTs [9, 28].

In this case, as the cyst was present in an unusual location, that is, the buccal soft tissue, IHC was applied to compare intraosseous KCOT and extraosseous KCOT in its Ki-67 staining properties.

Previous reports and observations reveal that, in conventional intraosseous KCOT, a larger number of suprabasal cells and very few basal cells take up the stain. But, in our case, a larger number of basal cells as compared to suprabasal cells showed positivity for anti-Ki-67 antibody.

This finding may question the general assumption that peripheral counterparts of central odontogenic lesions may exhibit less aggressive behaviour and may show lesser rates of recurrence. A larger number of cases need to be included to test this premise. Until then this will remain as a topic of speculation.

## 5. Conclusion

The diagnosis of KCOT of the jawbone is based purely on its characteristic histologic picture. However, in our case, similar findings were associated with a keratinizing cyst of the buccal mucosa. The locale for the predilection of this site is extremely rare, but the rationale for this topographic location can be explained by a theory of displaced and persistent dental lamina rests in the buccal mucosa during odontogenesis in the embryo. Therefore, though KCOT of the buccal mucosa represents an example of heterotopia, it is to be noted that the frequency of detection and identification of the peripheral variant of this pathology has risen. As our case is the 7th one to be reported till date, we propose that PKCOT deems recognition as a separate entity or as an extragnathic (soft tissue/mucosal) counterpart of the central (intraosseous) KCOT.

## Figures and Tables

**Table 1:** List of all the reported cases of KCOT in the buccal mucosa.

Case number	Age/sex	Treatment	Recurrence	Reference
1	59/M	Excision	—	Precheur and Krolls, 2009 [4]
2	60/M	Enucleation	No	Ide et al., 2010 [3]
3	16/M	—	—	Ide et al., 2010 [3]
4	74/M	Extirpation	No (4 yrs)	Yamamoto et al., 2013 [1]
5	37/M	Excision	No (12 mo)	Kaminagakura et al., 2013 [2]
6	52/M	Excision	No	Grobe et al., 2012 [5]
7	62/M	Excision in toto	No (2 yrs)	Present case
